# From the Proteome to Therapeutics: A Multi‐Database Approach to Drug Discovery in Periodontitis—An Exploratory Pilot Study

**DOI:** 10.1111/jcpe.70129

**Published:** 2026-05-04

**Authors:** Taisir Bozo, Annette Murr, Birte Holtfreter, Sebastian‐Edgar Baumeister, Peter Meisel, Uwe Völker, Werner Weitschies, Henry Völzke, Philipp Kanzow, Elke Hammer, Manuela Gesell Salazar, Thomas Kocher

**Affiliations:** ^1^ Department of Restorative Dentistry, Periodontology and Endodontology University Medicine Greifswald Greifswald Germany; ^2^ Department Functional Genomics, Interfaculty Institute for Genetics and Functional Genomics University Medicine Greifswald Greifswald Germany; ^3^ Institute of Health Services Research in Dentistry University of Münster Münster Germany; ^4^ German Centre for Cardiovascular Research (DZHK), Partner Site Greifswald Greifswald Germany; ^5^ Centre of Drug Absorption and Transport, Department of Biopharmaceutics and Pharmaceutical Technology, Institute of Pharmacy University of Greifswald Greifswald Germany; ^6^ Institute for Community Medicine University Medicine Greifswald Greifswald Germany

**Keywords:** data bank, drug discovery, periodontitis, proteomics, saliva

## Abstract

**Introduction:**

This explanatory pilot study presents a workflow to identify approved drugs, which could be repurposed for periodontitis therapy using salivary proteomics combined with drug‐target database screening.

**Methods:**

Proteomic analyses of saliva using LC–MS/MS were conducted in two independent settings: a cohort (sub‐study I, *N* = 187) and case–control (sub‐study II, *N* = 72). Statistical analyses were performed using both stringent and lenient criteria. Under the stringent (lenient) criteria, proteins were considered potential targets if they showed a *q*‐value < 0.05 (< 0.1) in the cohort study and a *p*‐value < 0.05 together with an absolute fold change > 1.5 (1.3) in the case–control study. Four databases were searched to assess whether these proteins are known targets of approved drugs.

**Results:**

In the stringent (lenient) analysis one (26) proteins were associated with periodontitis in both studies, of which one (nine) were reported drug targets and one (seven) of those were potential drug targets in periodontitis. Target screening in databases identified 3/11 approved drugs, with eight of them having potential for repurposing for periodontal treatment. Several of these drugs (e.g., HDAC inhibitors, sivelestat, compstatin derivatives, auranofin, artenimol and bortezomib) have already shown efficacy in preclinical models of periodontal host modulation.

**Conclusion:**

This exploratory pilot study provided insights into potential therapeutic targets and potential drugs for periodontitis treatment. This workflow may help to identify approved drugs that could be repurposed for periodontal treatment.

## 1 Introduction

Periodontitis is one of the most common diseases worldwide. Severe periodontitis affects over 1 billion people globally, with an age‐standardized prevalence of 12.5% (Nascimento et al. 2024) and approximately one‐third of permanent teeth in industrialized countries are extracted due to periodontitis. It is a chronic inflammatory disease, which is instigated by bacteria and characterized by destruction of the tooth‐supporting apparatus including periodontal ligament and alveolar bone (Kinane et al. 2017). Even though bacteria are required, the resident dysbiotic biofilm communities are insufficient to cause periodontitis (Hajishengallis et al. 2016). The inflammatory response of the host to the microbial assault is primarily responsible for the breakdown of periodontal tissue (Cekici et al. 2014; Zhang et al. 2024).

Salivary proteins originate from both local and systemic sources. Locally derived proteins are secreted predominantly by the major and minor salivary glands, gingival crevicular fluid (GCF) and oral epithelial cells. GCF, in particular, contains a high concentration of immune‐related proteins, inflammatory mediators and tissue breakdown products that directly reflect the host response to the subgingival biofilm. Systemically derived proteins reach the saliva through passive diffusion or active transport from the bloodstream into the salivary glands or via the GCF. Their elevated levels in periodontitis likely reflect a systemic acute‐phase response, which may be amplified by local periodontal inflammation. Mass spectrometry (MS)‐based proteomics is one method of quantifying these biomarkers, disentangling etiologic pathways or identifying biomarkers (Hussein and Kishen 2022). A systemic review of MS‐based saliva proteomics reported about 100 differentially abundant proteins between periodontitis‐affected and healthy subjects, and notably complement C3, neutrophil defensin, profilin‐1 and S100‐P were consistently replicated (Hu and Leung 2023).

Most pharmacological targets are proteins, which are causal in disease aetiology. After a pharmacological target has been identified in drug discovery, a drug screening procedure can help to identify appropriate therapeutic molecules (Hukerikar et al. 2024). Since proteins from the crevicular fluid are also present in saliva, saliva proteomics allows an unbiased approach to search for pharmacological targets of periodontitis.

Drug repurposing seeks new therapeutic indications for existing drugs beyond their original medical purpose, accelerating development timelines compared to traditional drug discovery by leveraging established pharmacological knowledge bases rather than de novo compound design, thereby facilitating the introduction of new therapeutic options (Abdelsayed et al. 2022; Pushpakom et al. 2019).

To date, periodontal therapy is mainly based on professional mechanical plaque removal and scaling, but there is an ongoing search for pharmacological therapy, modulating the local immunoinflammatory response. Research is attempting to identify potential drug targets based on the key molecules involved in the immune crosstalk of periodontitis (Ferra‐Canellas and Garcia‐Sureda 2024). Previous drug discovery has been based on prior etiological knowledge of drugs that act on known pathways. Many of these have been investigated in mechanistic animal studies.

To our knowledge, only a few studies have been conducted to unbiasedly search for potential drug targets for periodontal therapy (Alayash et al. 2026). Unbiased salivary proteomics can discover targets that can be screened in databases for their suitability as potential drug targets for periodontal therapy (Deng et al. 2025). First, we associated protein abundances with different periodontal variables in a cohort study and performed a replication in a case–control study to increase robustness. Second, only replicated proteins were searched in four databases as potential drug targets to identify approved drugs. The overall aim of our investigation was to present a workflow based on saliva proteomics to search for repurposed drugs for the treatment of periodontitis.

## 2 Materials and Methods

Details on the Studies of Health in Pomerania (SHIP), periodontal examinations, saliva sampling, LC–MS/MS analyses, covariates and statistical analyses are provided in the Data S1.

### 2.1 Study Design

#### 2.1.1 Sub‐Study I

In total, 187 nondiabetic participants (22–79 years) were randomly drawn from the first 1000 participants of the SHIP‐TREND‐0 cohort for saliva proteome assessments (Murr et al. 2017). Periodontal data were available for 180 participants. This study demonstrated that cohorts of approximately 150–200 individuals were sufficient to detect moderate‐to‐strong associations between protein abundance and continuous parameters.

#### 2.1.2 Sub‐Study II

Periodontally healthy controls and periodontitis cases within the 35–44 and 55–64 age groups were selected primarily from SHIP‐START‐2 and supplemented as necessary from SHIP‐TREND‐0. Exclusion criteria included smoking, diabetes and having fewer than 10 permanent teeth. Periodontally healthy controls were defined as those having bleeding on probing (BOP) < 30% and maximum probing depth (PD) ≤ 3 mm. Periodontitis cases were defined using study‐specific criteria, reflecting established SHIP protocols, as individuals with BOP > 10%, PD ≥ 5 mm at ≥ 2 sites and PD ≥ 4 mm at ≥ 40% of teeth. These criteria predate the 2017/2018 classification system and were retained to ensure consistency within the SHIP framework and comparability with earlier proteomic analyses. Of the eligible controls and cases, 20 cases and 20 age‐ and sex‐matched controls in the 35–44 age group, and 20 cases and 20 age‐ and sex‐matched controls in the 55–64 age group were selected. As proteomics measurements were not feasible for some samples, the final sample sizes were 20/17 for the 35–44 age group and 20/15 for the 55–64 age group, periodontitis cases versus healthy controls, respectively. There was no overlap of participants between sub‐studies I and II. Group sizes of 20 per disease status and age category were chosen to allow age‐ and sex‐matching, and to balance statistical power with feasibility constraints imposed by sample availability, financial limitations and the labour‐intensive nature of LC–MS/MS proteomic processing.

### 2.2 Protein Selection and Drug Target Screening

For protein selection, we performed two different analyses, the first analysis using stringent criteria and the second analysis using lenient criteria. In the first analysis of the cohort study (sub‐study I), proteins were required to be associated with at least two periodontal parameters (the percentage of sites with BOP (%BOP), the percentage of sites with probing depth ≥ 4 mm (%PD4+), mean PD and the cumulative sum of all PD values) at a false discovery rate‐adjusted *q*‐value < 0.05. In the case–control study (sub‐study II), independent replication was defined by *p*‐value < 0.05 and an absolute fold change (FC) > 1.5 between periodontally healthy controls and periodontitis cases within either the younger (35–44 years) or the older age group (55–64 years). To be included in the second, lenient analyses, the proteins had to fulfil the following requirements: (i) sub‐study I: *q* value < 0.1 in the linear regression analysis for any of the four periodontal variables, (ii) sub‐study II: *p* value < 0.05 and *abs*(FC) > 1.3 in at least one age group.

The two sub‐studies employed different proteomic platforms, with raw data analysed separately using consistent workflows and study‐specific false discovery rates (FDR). Results were merged only for replicated protein associations to limit platform‐specific bias, consistent with standard comparative proteomics approaches (Aebersold and Mann 2016; Altelaar et al. 2013; Merl‐Pham et al. 2019). In sub‐study I, protein identification used a 1% protein‐level FDR, while sub‐study II applied a 4% protein ion‐level FDR, following default Progenesis QI settings (v4.2; Nonlinear Dynamics, p. 51) and vendor‐recommended workflows (Waters Corporation 2017). Because FDR control differed in level and software framework, the nominal thresholds are not directly comparable.

### 2.3 Targets and Drug Banks

To identify druggable targets, protein targets were queried across the Therapeutic Target Database (TTD), Open Targets (OT) and DrugBank (DB). In addition, Perplexity was used to systematically query the biomedical literature using disease‐ and target‐specific prompts (e.g., “Which drugs could be used for anti‐inflammatory diseases targeting protein XY?”). Retrieved information was manually reviewed to ensure biological relevance and contextual consistency. Identified drug‐target associations were subsequently filtered according to therapeutic area and biological plausibility. Only drugs targeting host‐related pathways were considered, whereas compounds affecting the microbiome were excluded. The results from curated databases and literature‐based text mining were compiled to provide a comprehensive overview of proteins linked to approved drugs.

## 3 Results

### 3.1 Sub‐Study I

**FIGURE 1:**
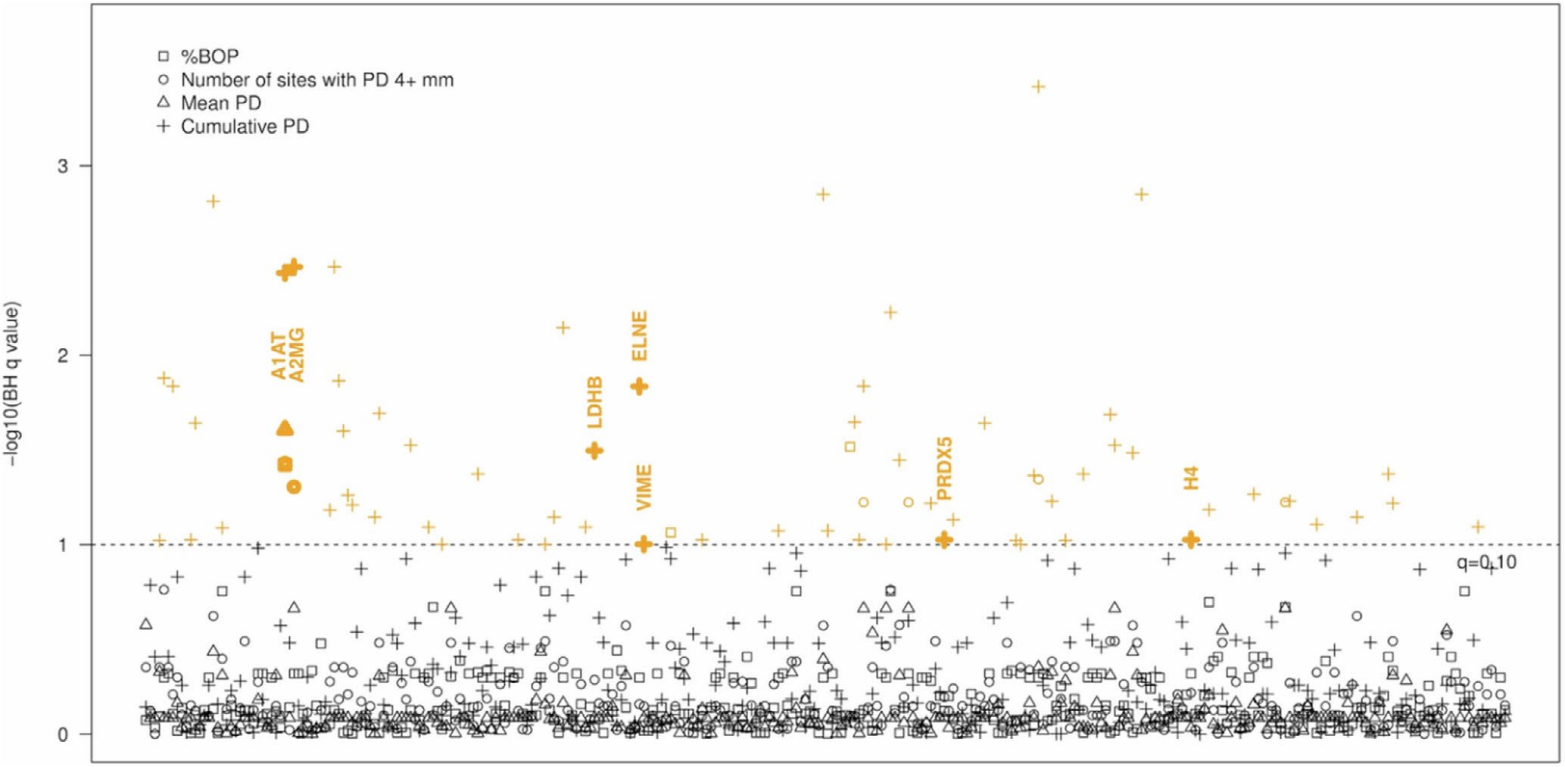
Sub‐study I: Manhattan plot of q values (−log10 transformed) for 304 quantified proteins, out of which 67 were significantly (*q* values < 0.1) associated with at least one of the periodontal variables. Proteins (*N* = 67) identified as potential drug target are highlighted in yellow and important target proteins (see section below) are labelled with their Uniprot entry names. BOP, bleeding on probing; PD, probing depth.

**FIGURE 2:**
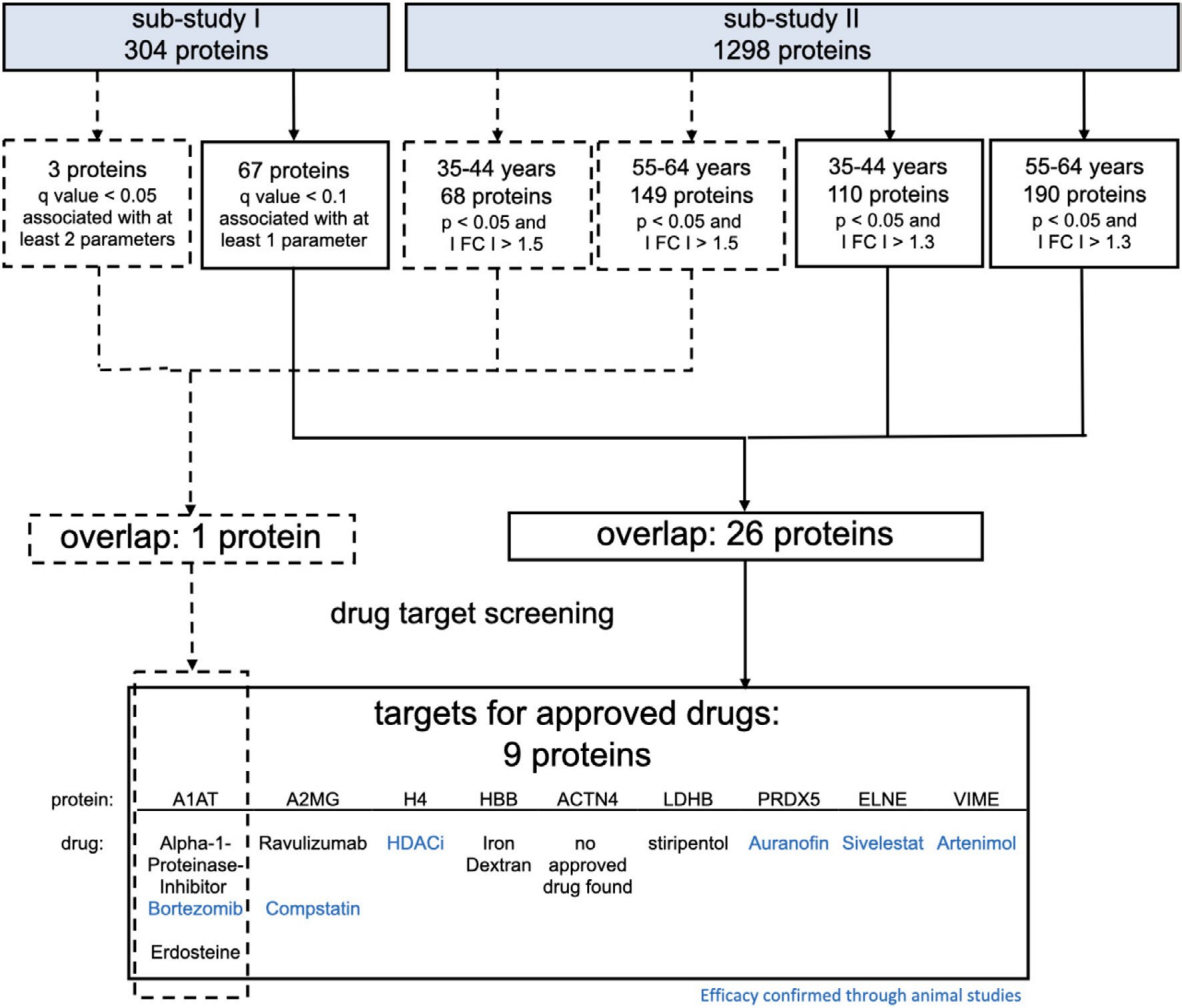
Flowchart summarizing the lenient (solid lines) and stringent (dashed lines) analytical pipelines applied to sub‐study I (304 proteins) and sub‐study II (1298 proteins) for drug‐target identification. The lenient analysis identified nine targets of approved drugs, while the stringent analysis highlighted A1AT as the only consistently replicated protein.

Participant characteristics are provided in Table 1 and the Data S1. Of 304 quantified proteins, 67 proteins were significantly associated with at least one periodontal variable (Figure 1). Applying the stringent criteria, three proteins were significantly associated with at least two periodontal variables (Figure 2). A2MG was associated with two periodontal variables (PD ≥ 4 mm, cumPD); serpin B5 was associated with three periodontal variables (%BOP, PD ≥ 4 mm, cumPD); and Alpha‐1‐antitrypsin (A1AT) was associated with all periodontal variables (Table S1). Applying the lenient criteria, 67 proteins were significantly associated with at least one of the periodontal variables. Two proteins were only associated with BOP (THIO, UBA1) or PD ≥ 4 mm (CALR, AHNAK), respectively. All other proteins were only associated with cumPD.

**TABLE 1:** Characteristics of the sub‐study I sample (*N* = 180) and of periodontally healthy controls and periodontitis cases within age groups in sub‐study II.

	Sub‐study I *N* = 180	Sub‐study II *N* = 72					
		35–44 years			55–64 years		
		Periodontally healthy controls	Periodontitis cases	*p*	Periodontally healthy controls	Periodontitis cases	*p*
Number of subjects	180	17	20	—	15	20	—
Age, years	49.6 ± 14.1	39.9 ± 3.2	40.3 ± 3.1	0.678	60.5 ± 3.3	59.0 ± 2.8	0.126
Male Sex	51.1%	47.1%	50.0%	0.858	46.7%	50.0%	0.845
School education							
< 10 years	8.9%	0.0%	10.0%		20.0%	20.0%	
10 years	54.4%	76.5%	80.0%		40.0%	60.0%	
> 10 years	36.7%	23.5%	10.0%	0.253	40.0%	20.0%	0.393
Smoking status							
Never smoker	39.4%	58.8%	30.0%		40.0%	50.0%	
Former smoker	35.6%	41.2%	70.0%	0.078	60.0%	50.0%	0.557
Current smoker	25.0%	—	—	—	—	—	—
Body Mass Index, kg/m^2^	27.5 ± 4.3	24.7 ± 2.6	28.8 ± 5.0	0.024	27.5 ± 4.8	30.3 ± 5.8	0.162
Haemoglobin A1c, %	5.0 ± 0.4	5.2 ± 0.4	5.1 ± 0.6	0.657	5.3 ± 0.4	5.4 ± 0.5	0.750
Protein concentration, μg/μL	—	3.97 ± 1.70	4.27 ± 1.96	0.461	4.71 ± 3.34	4.97 ± 3.04	0.816
Periodontal variables							
Number of teeth	22.6 ± 6.3	25.9 ± 1.4	24.7 ± 2.5	0.090	22.6 ± 3.8	21.0 ± 4.7	0.254
BOP Score, %	21.8 ± 23.5	2.7 ± 3.6	47.4 ± 20.2	< 0.001	3.5 ± 3.6	56.5 ± 20.6	< 0.001
Mean PD, mm	2.45 ± 0.52	2.02 ± 0.20	3.03 ± 0.35	< 0.001	2.09 ± 0.22	3.07 ± 0.38	< 0.001
Maximum PD, mm	4.46 ± 1.58	3.0 ± 0	6.45 ± 1.23	—	3.0 ± 0	6.15 ± 1.09	—
Cumulative PD, mm	109.0 ± 32.9	103.8 ± 14.2	148.0 ± 23.3	< 0.001	93.1 ± 22.7	129.1 ± 36.3	< 0.001
Percentage of sites with PD ≥ 5 mm, %	4.2 ± 9.4	0 ± 0	6.2 ± 3.7	—	0 ± 0	5.3 ± 4.2	—
Percentage of teeth with PD ≥ 4 mm, %	26.7 ± 30.5	0 ± 0	61.9 ± 15.6	—	0 ± 0	70.3 ± 18.9	—
Number of sites with PD ≥ 4 mm	4.3 ± 5.8	0 ± 0	12.8 ± 4.9	—	0 ± 0	13.3 ± 7.1	—
Mean CAL, mm	2.01 ± 1.41	1.40 ± 0.53	2.80 ± 0.86	< 0.001	2.05 ± 0.65	3.87 ± 0.82	< 0.001

*Note*: Data are presented as mean ± standard deviation or percentage. *p* values were retrieved from Mann–Whitney *U* tests or Chi‐squared tests.

Abbreviations: BOP, bleeding on probing; CAL, clinical attachment level; PD, probing depth.

Functional enrichment analyses were performed to support biological interpretation rather than discovery, using g:Profiler for function of molecules (GO terms) and involvement in biological pathways (Reactome). Enrichment results consistently highlighted immune and neutrophil‐related pathways and protease activity (Figure S1 (left column each), Table S2).

### 3.2 Sub‐Study II

**FIGURE 3:**
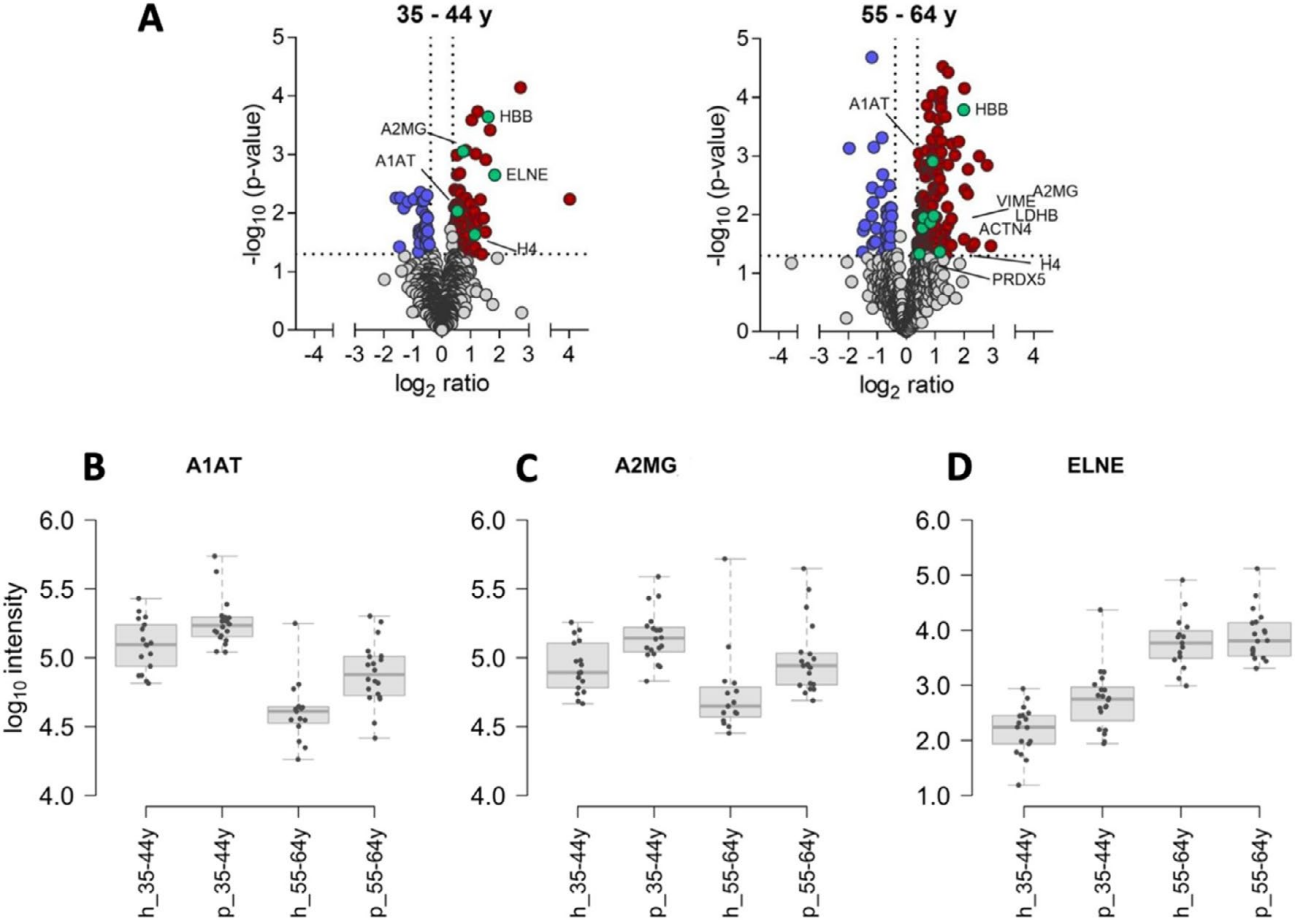
(A) Sub‐study II: Volcano plots show the differential level of up and downregulated 1298 proteins in the two age groups. Upregulated proteins appear on the right and downregulated on the left side. The *x*‐axis represents the fold change in protein abundance between the periodontally healthy and the diseased case group. The *y*‐axis represents the statistical significance of the change as the negative logarithm of the *p*‐value. In this graph, all proteins with a *p* value ≤ 0.05 and FC > I1.3I are coloured in red and blue, and potential drug targets are shown in green colour and labelled with their Uniport entry name. (B–D) Boxplots showing the log10 intensity distribution of selected proteins (A1AT, A2MG, ELNE) across periodontally healthy controls (h) and periodontitis cases (p) in younger (35–44 years) and older (55–64 years) age groups.

Participant characteristics are provided in Table 1 and the Data S1. Of 1298 quantified proteins, several proteins, like A2MG, A1AT, PRTN3, HBB, HBA and ABRAL, showed significantly higher levels in periodontitis cases in both age groups, while other proteins such as TGM1, SHAN3 and ARP2 exhibited reduced abundance (Table S3; Figure 3A). For some proteins (e.g., A1AT, neutrophil elastase [ELNE]), levels differed significantly across disease and age groups (Figure 3B,D). By contrast, the differences related to periodontitis were rather similar for A2MG in both age groups (Figure 3C). Applying the stringent criteria, 68 and 149 differed significantly between periodontally healthy controls and periodontitis cases in the younger and the older age group, respectively (Figure 2). Applying the lenient criteria, 110 and 190 proteins differed significantly between periodontally healthy controls and periodontitis cases in the younger and the older age group, respectively.

**FIGURE 4:**
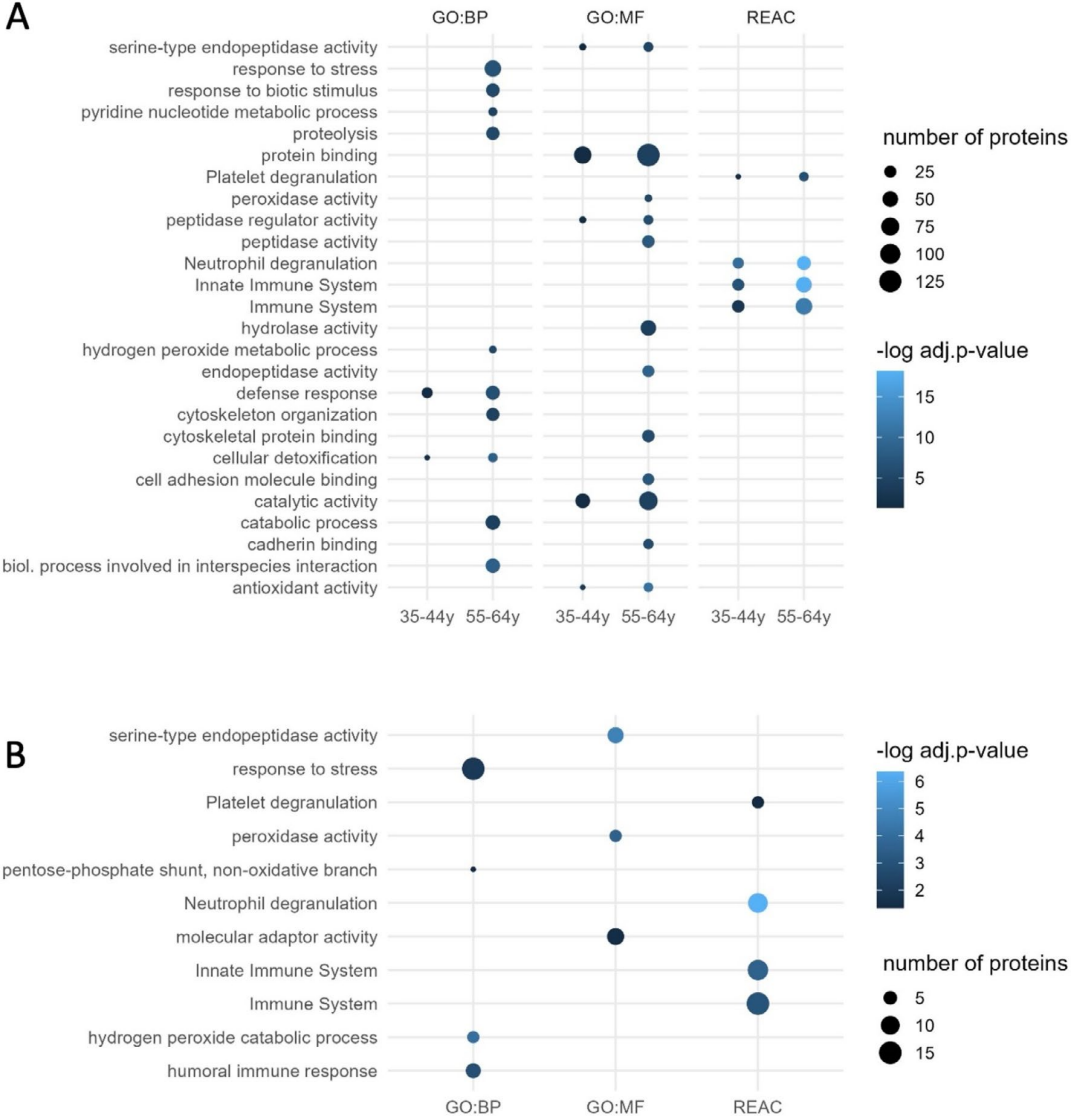
Results of protein enrichment analyses for interpretation of protein sets regarding biological processes (GO:BP), molecular functions (GO:MF) and involvement in pathways (Reactome (REAC)). (A) Results of study II stratified by age group. Data were selected from the TOP50 categories (FDR < 0.05) revealed in g:Profiler analyses. (B) Results of enrichment analyses of the overlap found across both sub‐studies (*n* = 26 proteins). Terms presented by the same set of proteins are clustered. The size of each dot is proportional to the number of proteins associated with a given term, while the dot colour represents the significance of the enrichment, shown as the negative log‐transformed *p*‐value.

Enrichment analyses of proteins differentially abundant between periodontitis cases and healthy controls identified functional patterns largely consistent with those observed in sub‐study I (Figure 4; Figure S1; Table S2), despite differences in study design and age stratification. Enriched GO:BP terms reflected sustained immune activation and stress responses, including pathways linked to neutrophil function, oxidative‐stress regulation and cellular detoxification. GO:MF categories related to antioxidant activity and proteolysis further emphasized the involvement of redox balance and tissue remodelling. REAC pathway analysis confirmed enrichment of immune system–related pathways across both age groups, with minor variations in pathway prominence, supporting biological convergence between case–control status and continuous measures of periodontal parameters.

### 3.3 Protein Selection for Drug Target Screening

Applying the more stringent criteria only A1AT was consistently associated with periodontitis across both sub‐studies. The joint evaluation of the two sub‐studies according to the lenient criteria yielded 26 proteins (Figure 2; Table 2). While suprabasin (SBSN) demonstrated a negative association with periodontitis, the remaining proteins exhibited a positive association. For 15 proteins (A1AT, A2MG, CAP7, H4, HBB, PRTN3, GDIR2, ACTN4, PERM, LDHB, MMP8, PRDX5, PDIA3, ELNE, SBSN) the direction of association remained consistent across all periodontal variables of sub‐study I and within both age groups of sub‐study II.

**TABLE 2:** Overview of the proteins identified in (i) sub‐study I with a *q* value of less than 0.1 (bold) and (ii) sub‐study II with an absolute fold change of more than 1.3 and a *p* value of < 0.05 (bold).

Entry name	Protein name	Sub‐study I, β coefficients *N* = 180				Sub‐study II abs(FC) values *N* = 72	
		%BOP	PD ≥ 4 mm	Mean PD	Cumulative PD	35–44 years (*N* = 17/20)	55–64 years (*N* = 15/20)
ACTN4	Alpha‐actinin‐4	0.175	0.013	0.095	**0.007**	1.24	**1.47**
CAP7	Azurocidin	0.341	0.022	0.279	**0.006**	**1.95**	**1.36**
LDHB	L‐lactate dehydrogenase B chain	0.063	0.018	0.172	**0.006**	1.05	**1.92**
H4	Histone H4	0.529	0.021	0.056	**0.005**	**2.20**	**2.22**
PRTN3	Myeloblastin	0.545	0.020	0.121	**0.005**	**2.36**	**2.55**
PERM	Myeloperoxidase	0.140	0.019	0.168	**0.005**	1.06	**2.21**
MMP8	Neutrophil collagenase	0.194	0.027	0.271	**0.005**	1.19	**2.31**
PGRP1	Peptidoglycan recognition protein 1	0.186	0.019	0.212	**0.005**	−1.01	**2.30**
PDIA3	Protein disulfide‐isomerase A3	0.138	0.014	0.127	**0.005**	1.17	**1.58**
GDIR2	Rho GDP‐dissociation inhibitor 2	0.025	0.020	0.186	**0.005**	**1.78**	**2.01**
ELNE	Neutrophil elastase	0.286	0.009	0.028	**0.004**	**3.55**	1.27
A2MG	Alpha‐2‐macroglobulin	0.280	**0.018**	0.213	**0.003**	**1.67**	**1.79**
PRDX5	Peroxiredoxin‐5, mitochondrial	0.168	0.008	0.058	**0.003**	1.09	**1.36**
VIME	Vimentin	0.033	0.009	0.127	**0.003**	−1.04	**1.52**
A1AT	Alpha‐1‐antitrypsin	**0.242**	**0.011**	**0.137**	**0.002**	**1.46**	**1.88**
FIBA	Fibrinogen alpha chain	−0.229	0.008	0.053	**0.002**	−1.21	**1.38**
HBB	Haemoglobin subunit beta	0.289	0.009	0.095	**0.002**	**3.03**	**3.94**
CAPG	Macrophage‐capping protein	−0.056	0.010	0.112	**0.002**	1.24	**1.44**
TKT	Transketolase	0.186	0.006	−0.003	**0.002**	**1.47**	**2.18**
VTDB	Vitamin D‐binding protein	0.150	0.003	−0.001	**0.002**	−1.10	**1.62**
LEG3	Galectin‐3	0.092	0.001	−0.011	**0.001**	1.23	**1.31**
SPB10	Serpin B10	−0.01	−0.003	−0.013	**−0.001**	1.13	**1.84**
PRDX4	Peroxiredoxin‐4	0.003	−0.009	−0.160	**−0.002**	−1.04	**2.55**
ROA2	Heterogeneous nuclear ribonucleoproteins A2/B1	0.110	−0.001	−0.002	**−0.002**	1.05	**1.49**
RPE	Ribulose‐phosphate 3‐epimerase	−0.335	−0.018	−0.176	**−0.003**	n.a.	**2.57**
SBSN	Suprabasin	−0.194	−0.022	−0.114	**−0.004**	**−1.47**	**−2.27**

*Note*: For sub‐study I, coefficients from linear regression models are shown (β with 95% confidence interval per 1 standard deviation increase of the periodontal variable). For sub‐study II, absolute fold change (FC) values are presented. We have underlined the proteins for which we identified drug targets. This table lists proteins in descending order based on the β coefficient for cumulative PD.

Abbreviations: BOP, bleeding on probing; FC, fold change; PD, probing depth.

Analysing the reduced set of 26 proteins resembled the enrichment terms described above (Figure 4B; Table S4). In both analyses, the highest specificity was found for immune system related categories (PGRP1, ELNE, FIBA, CAP7, PRTN3, LEG3, CAPG) followed by hydrogen peroxide catabolic process/cellular oxidant detoxification (PERM, PRDX4, PRDX5, SPB10, HBB, A1AT).

### 3.4 Drug Discovery

We found the following potential drugs for the A1AT target: alpha‐1‐proteinase inhibitor, bortezomib and erdosteine. For the lenient analysis out of the 26 proteins, nine have already been reported as drug targets (ACTN4, LDHB, H4, ELNE, A2MG, PRDX5, VIME, A1AT, HBB), seven of which are drug targets for periodontitis (excluding ACTN4 and HBB) (Figure 2; Table 3). These are targets of 11 approved drugs (stiripentol, HDACi, sivelestat, ravulizumab, derivatives of compstatin, auranofin, artenimol, alpha‐1‐proteinase inhibitor, bortezomib, erdosteine and iron dextran). Eight of these drugs (excluding stiripentol, erdosteine and iron dextran) exhibit anti‐inflammatory properties. Six of these compounds (HDACi, sivelestat, derivative of compstatin, auranofin, artenimol, bortezomib) have already shown efficacy in periodontal host modulation in preclinical animal studies, while for a compstatin derivative efficacy was demonstrated in a Phase 2 clinical trial in humans.

**TABLE 3:** Compilation of the protein targets, potential drugs and the respective diseases for which the drug is used, as well as the pharmacological effect of the drug. The selection of targets was based on a search of the following databases: The Therapeutic Target Database, Open Targets, DrugBank and Perplexity.

Protein	Physiological function of protein	Drug	Drug target	Drug mechanism	Disease	Periodontal investigation	Source^a^
ACTN4 (Alpha‐actinin‐4)	Cytoskeletal organization; Cell motility and migration	No approved drug					
LDHB (l‐lactate dehydrogenase B)	Energy production Redox balance; Glucose homeostasis Antioxidant defence	Stiripentol	LDHB inhibits enzyme activity	LDHB inhibitor: primarily an anti‐convulsant without clear anti‐inflammatory effects.	Epilepsy		3
Histone H4 (H4)	Chromatin organization DNA repair and stability; Gene regulation; Immune response and inflammation Stress response	HDACi inhibitor (vorinostat, panobinostat)	Removes acetyl groups	Blocks deacetylation, increasing acetylation of histones vorinostat showed in rodent models of arthritis and colitis anti‐inflammatory effects	T cell lymphomas Multiple myeloma	Suppress periodontal bone loss in rodents (Cantley et al. 2017)	4
ELNE (Neutrophil elastase)	Immune regulation Dysregulated expression leads to chronic inflammation	Sivelestat	Selective inhibitor of human neutrophil elastase	Reduce inflammation by preventing infections.	Lung injury Postoperative care cardiopulmonary surgery	In vitro Sivelestat inhibits the leukotoxin of Aggregatibacter actinomycetem‐comitans (Hiyoshi et al. 2019)	3
A2MG (Alpha‐2 macroglobulin)	Protease inhibition; Immune modulation; Removal of damaged proteins; Keystone in responding to tissue injury or infection.	Ravulizumab	Complement C5 protein (C5)	Inhibits C5, reducing complement‐mediated inflammation	Neuromyelitis optica; Paroxysmal nocturnal haemoglobinuria		1
	A2MG and C3/C5 share structural and evolutionary relationships. They contribute to the broader immune response	Derivatives of compstatin (pegcetacoplan)	Complement C3 (CO3)	Inhibits complement protein C3, reducing complement‐mediated inflammation	Non‐Hodgkin lymphoma; Breast cancer Macula degeneration	Amy‐101 (derivatives compstatin) inhibits pre‐existing chronic periodontal inflammation in humans (Hasturk et al. 2021)	1
PRDX5 (Peroxiredoxin‐5, mitochondrial)	Antioxidant defence; Bone homeostasis Regulation of inflammation	Auranofin	Targets thioredoxin reductase	Inhibits inflammatory pathways (e.g., NF‐κB activation) and reduces pro‐inflammatory cytokines	Rheumatoid arthritis	Supress periodontal bone loss in rodents (Valerio et al. 2019)	3
VIME (Vimentin)	Cytoskeletal structure; Cell motility and migration; Organelle positioning Endothelial regulation Wound healing; Immune response	Artenimol	Targets various cellular proteins	Antimalarial, binds to a large number of targets; protects against bone loss.	Malaria	In vitro antimicrobial activity against the periodontopathic bacteria; anti‐inflammatory (Kim, Choi, et al. 2015) In rodents targeting both bone formation and resorption processes (Zhou et al. 2016)	3
A1AT (Alpha‐1 Antitrypsin)	Protease inhibition; Anti‐inflammatory action; Regulation of lymphocyte movement; Tissue protection	Alpha‐1‐proteinase inhibitor	Alpha‐1‐antitrypsin (SERPINA1)	Inhibition of neutrophil elastase and reduction of inflammation in lung tissues	Emphysema; Alpha‐1 antitrypsin deficiency		1, 3
		Bortezomib	Cationic trypsinogen (PRSS1) A1AT and PRSS1 interact	Does not directly target A1AT or PRSS proteins, but influences accumulation of misfolded proteins like A1AT through proteasome inhibition. It has anti‐inflammatory properties and can inhibit osteoclast activity and reduce bone resorption.	Mantle cell lymphoma; Multiple myeloma	In rodent models, bortezomib inhibits periodontal bone destruction (Jiang et al. 2017; Kim, Kang, et al. 2015)	1
		Erdosteine	Pancreatic elastase 1 (CELA1)	Maintains A1AT function Mucolytic with antioxidant and mild anti‐inflammatory effects in airway diseases; does not target specific proteins directly; has multifaceted action	Chronic obstructive pulmonary disease Bronchitis		1
HBB (Haemoglobin subunit beta)	Oxygen binding and transport	Iron dextran		Haemoglobin and Erythropoiesis: Transferrin delivers iron to the bone marrow, where it is incorporated into haemoglobin during red blood cell production. This helps restore normal RBC levels in iron‐deficient patients (iron dextran promotes both inflammation and bone resorption rather than preventing them!)			3, 4

^a^ 1: Therapeutic Target Database; 2: Open Targets Platform; 3: Drug bank; 4: Perplexity.

## 4 Discussion

Our exploratory pilot study successfully identified one druggable target in the stringent analysis and nine druggable targets in the lenient analysis with approved compounds with potential relevance for periodontitis therapy. Given the exploratory nature of the study and the limited sample size, both stringent and lenient statistical thresholds were applied to avoid the premature exclusion of potentially relevant targets.

Based on the well‐established collection with Salivette, saliva was obtained reproducible in terms of mucosal protein content and microbial protein depletion. The use of different proteomic technologies provides a form of cross‐platform validation, as only proteins reproducibly associated with periodontal status across independent analytical settings were retained. This design minimizes platform‐specific biases and strengthens confidence in replicated protein associations. The much higher number of proteins quantified in saliva samples of sub‐study II shows the potential of the more modern data‐independent acquisition (LC–MS^E^) method in comparison with the MS results obtained in sub‐study I by data‐dependent acquisition.

According to the stringent analysis, only A1AT was consistently associated with periodontitis. A1AT modifies periodontal tissue destruction via regulation of neutrophil serine proteases and inflammatory mediators (Lechowicz et al. 2020). The 26 proteins found in the lenient analysis (Table 2; Figure 2) are highly consistent with the known pathogenesis of periodontitis, as the majority of the overlapping proteins have previously been implicated in periodontal inflammation or host‐response mechanisms. Functional analysis revealed enrichment in pathways associated with defence response, immune system, neutrophil and platelet degranulation—processes that align well with established mechanisms of periodontal inflammation (Figure S1).

Some of the 26 proteins are well studied (e.g., myeloperoxidase [MPO], neutrophil elastase) with respect to periodontitis, while others remain unexplored and may be false positives (ACTN4, transketolase, etc.) (Bostanci et al. 2018; Hu and Leung 2023). Seven out of the nine druggable proteins are involved in inflammation, oxidative‐stress response or bone health. Locally derived proteins in our dataset include ELNE, MPO and S100 calcium‐binding proteins (S100‐A8, S100‐A9), all of which are released from neutrophil granules into periodontal pockets during active inflammation. Systemically derived proteins include A1AT, alpha‐2‐macroglobulin (A2MG) and complement component C3, which are synthesized mainly in the liver but can also be detected in saliva. Whether this distinction is relevant for assessing their therapeutic potential in periodontitis remains open to discussion. The nine druggable targets among these 26 proteins are summarized in Table 3. Those proteins have prominent functions in the detoxification/production of reactive oxygen species (PRDX5), thereby supporting the defense against microbial infection (Kang et al. 2005; Rhee et al. 2012). On the other hand, neutrophil elastase (ELNE) reflects the immune and stress response characteristic of periodontal inflammation, which contributes to periodontal tissue destruction (Hiyoshi et al. 2022; Vitkov et al. 2017). Altogether, these protein signatures converge on central biological processes such as cell migration, metabolic regulation, protease/antiprotease balance, chromatin remodelling, oxidative‐stress response and immune defense, each of which contributes to periodontal tissue breakdown and repair.

Drug screening relied on established databases, as inherent to drug repurposing. Novelty of our workflow lies in linking unbiased salivary proteomic signatures to pharmacologically actionable targets in periodontitis. The only significant protein of the stringent analysis was A1AT, which is a target for bortezomib, an approved proteasome inhibitor, which acts indirectly on A1AT by inhibiting proteasomal degradation pathways involved in the turnover of misfolded proteins (Goldberg 2012; Lechowicz et al. 2020). It prevents alveolar bone loss and inhibits osteoclast differentiation by interfering with RANKL‐ and LPS‐dependent signalling in a mouse model (Jiang et al. 2017; Kim, Kang, et al. 2015).

There are no studies that directly link ACTN4 to periodontitis, while LDHB plays a crucial role in energy metabolism and cellular balance (Brisson et al. 2016; Liang et al. 2016) and has been associated with periodontal disease (Ali et al. 2018; De La Pena et al. 2007). Together with A2MG and A1AT, LDHB reflects a functional cluster related to metabolic regulation and the protease/antiprotease balance characteristic of chronic inflammatory states (Ali et al. 2018; Vandooren and Itoh 2021). Stiripentol, which acts on LDHB, is primarily known for its anti‐convulsant properties and does not have anti‐inflammatory effects (DrugBank 2025). No study has been published in relation to periodontitis so far. Extracellular histone H4 plays a key role in inflammation by inducing cell death and activating immune responses (Lechowicz et al. 2020). It exacerbates inflammatory diseases, including periodontitis, by promoting Th17‐driven inflammation and bone loss (Jiang et al. 2017; Kim, Kang, et al. 2015). Its upregulation also implies epigenetic modulation through histone acetylation or methylation, which can influence the transcriptional response to bacterial and inflammatory stimuli (Hu and Fan 2025; Laberge et al. 2023). Histone deacetylase inhibitors (HDACi) such as vorinostat and Panobinostat (Perplexity 2025), already approved for cancer treatment, may have potential in periodontitis therapy by modulating inflammation and bone resorption (Cantley et al. 2017).

Neutrophil elastase (ELNE), a serine protease, primarily found in neutrophils, is released during inflammation and contributes to periodontitis by degrading extracellular matrix proteins and playing a role in NET formation (Hiyoshi et al. 2022; Vitkov et al. 2017). The enrichment of ELNE in our dataset corresponds to its established role in proteolytic tissue damage during periodontitis (Hiyoshi et al. 2022). Several inhibitors have been developed to regulate ELNE activity, including endogenous inhibitors such as α1‐proteinase inhibitors and synthetic inhibitors such as sivelestat. Sivelestat is clinically used in the treatment of acute lung injury and acute respiratory distress syndrome, where excessive neutrophil elastase activity contributes to tissue damage (Aikawa et al. 2011). In Addition, an in vitro study showed that sivelestat mitigated the release of ELNE from neutrophils stimulated with periopathogenic bacteria (Hiyoshi et al. 2019).

A2MG regulates inflammation and immune responses by inhibiting proteases and binding cytokines (Cuellar et al. 2016). It shares similarities with complement factors C3 and C4 in protease scavenging (Vandooren and Itoh 2021). The complement system provides multiple targets for pharmacological intervention, with C3 being particularly important. A compstatin derivative blocks C3 activity, preventing activation of the complement cascade and inflammation (Lamers et al. 2022; Zhang et al. 2026). Ravulizumab is a humanized monoclonal antibody that blocks C5 activation (Kulasekararaj et al. 2019). Locally applied Compstatin AMY‐101, an inhibitor of the complement component C3, appears to be a promising candidate drug for the adjunctive local treatment of human periodontitis (Hasturk et al. 2021).

PRDX5 and related antioxidant enzymes highlight oxidative‐stress pathways and redox regulation–major components of chronic periodontal inflammation (Kang et al. 2005; Rhee et al. 2012). This reinforces the view that oxidative stress and antioxidant defence represent pivotal, druggable processes in periodontitis (Ramya et al. 2024). Auranofin modulates cellular redox homeostasis via thiol‐ and thioredoxin‐dependent pathways, thereby indirectly affecting peroxiredoxin‐related antioxidant mechanisms (Roder and Thomson 2015). It reduced alveolar bone loss in a rat model of periodontitis (Valerio et al. 2019).

VIME, an intermediate filament protein in mesenchymal cells, is present in periodontal fibroblasts (Naruishi 2022). The presence and upregulation of VIME and ACTN4 suggest enrichment in pathways related to cell adhesion, migration and cytoskeleton organization—key processes in inflammation, wound healing and tissue remodelling (Guo et al. 2013; Shindo et al. 2023). Artenimol (dihydroartemisinin) has been shown to interact with vimentin and to modulate cytoskeleton‐associated and metabolic processes, contributing to oxidative‐stress–mediated effects (Roy et al. 2020). No data are currently available regarding its role in periodontitis.

HBB has been linked to periodontitis in several studies (Thaweboon et al. 2010; Torres et al. 2024), particularly in the context of inflammation and oxidative stress (Torres et al. 2024). Its increased abundance in our study may therefore indicate microvascular injury, a hallmark of chronic inflammation (Ansari Moghadam et al. 2022). Iron dextran, an approved iron replacement therapy used for the treatment of iron deficiency anaemia, was identified in the database screening due to its functional association with haemoglobin metabolism rather than a direct pharmacological targeting of HBB. However, further research revealed that iron overloaded with iron dextran likely led to elevated oxidative stress in bone tissue of mice (Tsay et al. 2010).

The observation that only a subset of identified proteins is linked to approved drugs is consistent with established drug repurposing studies, where druggable targets typically represent a minority of biologically relevant candidates due to pharmacological and structural constraints rather than methodological limitations (Dudley et al. 2011; Pushpakom et al. 2019). Large‐scale analyses of the human druggable genome suggest that approximately one‐fifth of protein‐coding genes are considered druggable (Finan et al. 2017).

Even though repurposed drugs have limited safety issues and side effects are tolerable within intended use, the potential side effects of periodontitis treatment could be too great. Besides, when used in the oral cavity as local agents, drug development faces different hurdles depending on the treatment phase of periodontitis. All identified drugs are administered systemically, but for use in prevention or for local treatment of teeth with a poor periodontal prognosis, toothpastes, mouthwashes or slow release devices are the treatment of choice. Toothpaste and mouthwash, however, have limited efficacy as drug delivery systems due to short contact time and poor tissue penetration (Szulc et al. 2018).

Limitations include small sample sizes, heterogeneous proteomic workflows and limited sensitivity for low‐abundance salivary proteins. We deliberately applied lenient thresholds to avoid premature exclusion of relevant targets but may have increased false negatives. The heterogeneity of periodontitis variables may limit comparability but also supports cross‐validation of signals across differing aspects of periodontal inflammation.

We cannot exclude that ACTN4 is a false positive among the 9 target proteins. The pilot study is exploratory and does not establish causality. However, experimental animal studies targeting H4, ELNE, VIME and PRDX5 demonstrated reduced periodontal inflammation and bone loss, supporting a causal contribution of these proteins to periodontal disease mechanisms (Table 3; Cantley et al. 2017; Hasturk et al. 2021; Hiyoshi et al. 2019; Jiang et al. 2017; Kim, Choi, et al. 2015; Kim, Kang, et al. 2015; Valerio et al. 2019; Zhou et al. 2016). Finally, the pipeline did not capture classical targets such as COX enzymes, consistent with its proof‐of‐concept nature rather than comprehensive pathway coverage. The aim was not to map known mechanisms but rather to demonstrate a proof‐of‐concept pipeline for drug discovery in periodontitis using unbiased proteomics. Candidate drugs differ substantially in feasibility and safety; therefore, future work should prioritize agents suitable for local delivery.

## 5 Conclusion

The strength of our work lies in its innovative approach to identify therapeutic targets for periodontitis by combining untargeted proteomics with drug database screening. Our exploratory pilot study is the first to use salivary proteomics not for biomarker discovery but to identify drug repurposing candidates for periodontal therapy. By analysing two independent sub‐studies, we identified 26 key proteins, among which nine may serve as potential drug targets, with seven specifically linked to inflammatory regulation. Our study serves as a proof‐of‐concept supporting this workflow by identifying eleven approved drugs, eight of which have potential for periodontal therapy. Despite limitations (e.g., sample size, periodontitis case definition, causal validation), it highlights proteomics‐based drug discovery as a promising strategy in periodontology.

## Author Contributions

T.B., T.K., A.M., B.H., M.G.S. and E.H. substantially contributed to the conception or design of the work. T.B., A.M., M.G.S., T.K., B.H. and E.H. contributed to the analysis or interpretation of data. T.B., T.K., B.H., M.G.S. and E.H. drafted the work. S.‐E.B., P.K., P.M., W.W., U.V. and H.V. revised the work critically for important intellectual content. All authors approved the final version of the manuscript and are accountable for all aspects of the work.

## Funding

SHIP is part of the Community Medicine Research Network of the University Medicine Greifswald, which is supported by the German Federal State of Mecklenburg‐West Pomerania.

## Ethics Statement

All SHIP studies were positively evaluated by the ethics committee of the University of Greifswald (SHIP‐START‐2: BB 39/08 issued on June 19th 2008; SHIP‐TREND‐0: BB 39/08a issued on September 3rd 2009). Written informed consent was obtained from all participants prior to their enrolment.

## Conflicts of Interest

The authors declare no conflicts of interest.

## Supporting information


**Figure S1:** Comparison of protein enrichment across different studies for biological processes (GO:BP), molecular functions (GO:MF) and Reactome (REAC) pathways. The dot plot visualizes the Top50 significantly enriched terms for biological processes (GO:BP), molecular functions (GO:MF) and Reactome (REAC) pathways. The size of each dot is proportional to the number of proteins associated with a given term, while the dot colour represents the significance of the enrichment, shown as the negative log‐transformed *p*‐value.
**Table S5:** Detailed LC–MS/MS and database search parameters for measurement and database search for sub‐study I (Murr et al., 2017).
**Table S6:** Parameters for measurement and database search for sub‐study II.
**Data S1:** Materials and methods.

**Table S1:** Detailed results from linear regression analyses for sub‐study I.

**Table S2:** Detailed results of protein enrichment analysis (GO and Reactome).

**Table S3:** Results from analysis of younger (33–45 years) and older (55–64 years) age groups in sub‐study II.

**Table S4:** Functional annotation of the 26 target proteins identified across both sub‐studies using g:profiler.

## Data Availability

Proteome data that support the findings of this study are openly available in ProteomeXchange at https://www.proteomexchange.org/, reference number PXD006367 and PXD068002. SHIP data are available from Forschungsverbund Community Medicine (CMR). Restrictions apply to the availability of these data, which were used under licence for this study. Data are available from https://transfer.ship‐med.unigreifswald.de/FAIRequest/login with the permission of Forschungsverbund Community Medicine.
