# Supplementary material for: From the Proteome to Therapeutics: A Multi‐Database Approach to Drug Discovery in Periodontitis—An Exploratory Pilot Study
Source: J Clin Periodontol. 2026 May 4;53(7):990–1004. doi: 10.1111/jcpe.70129 (PMC13263717; doi:10.1111/jcpe.70129)
Supplement: Supplementary file 1 — Figure S1: Comparison of protein enrichment across different studies for biological processes (GO:BP), molecular functions (GO:MF) and Reactome (REAC) pathways. The dot plot visualizes the Top50 significantly enriched terms for biological processes (GO:BP), molecular functions (GO:MF) and Reactome (REAC) pathways. The size of each dot is proportional to the number of proteins associated with a given term, while the dot colour represents the significance of the enrichment, shown as the negative log‐transformed p‐value. Table S5: Detailed LC–MS/MS and database search parameters for measurement and database search for sub‐study I (Murr et al., 2017). Table S6: Parameters for measurement and database search for sub‐study II. Data S1: Materials and methods. [file JCPE-53-990-s004.docx]

Supporting Information

**From the Proteome to Therapeutics:**

**A Multi-Database Approach to Drug Discovery in Periodontitis – an exploratory study**

Materials & methods

Study design

The Studies of Health in Pomerania (SHIP) are prospective population-based cohorts in northeast Germany. For SHIP-START-0, the sample was stratified in terms of age, sex and place of residence (Keil et al., 1988). A two-stage cluster design was adopted, yielding twelve five-year age strata (20-79 years) for both genders, each stratum including 292 individuals. A representative sample of 7,008 subjects with German citizenship aged 20-79 years was selected from the population registration offices. The net sample (without deceased and migrated persons) comprised 6265 eligible subjects, out of which 4308 individuals (response 68.8%) participated in baseline examinations between 1997 and 2001. Between 2002 and 2006 all participants were re-invited for an examination follow-up (SHIP-START-1), in which 3300 subjects took part (83.5% of all eligible subjects). The second follow-up examination (SHIP-START-2) was conducted eleven years later (2008–2012; N=2333). Five calibrated and licensed dentists performed the dental examinations. Calibration exercises were conducted every 6-12 months. In SHIP-START-2, intra-class correlations of 0.76-0.88 per examiner and an inter-class correlation of 0.74 for CAL were achieved. For PD, intra-class correlation was 0.70-0.78 per examiner and inter-class correlation was 0.70.

For SHIP-TREND, a random sample of 10,000 adults aged 20-79 years was drawn from the central resident’s registration office of the federal state of Mecklenburg/Western Pomerania (Volzke et al., 2022). The sample was stratified in terms of age, sex and place of residence. Final sample size amounted to 4,420 participants (response 50.1%). Baseline examinations were conducted between 2008 and 2012. Every 6–12 months, all ﬁve examiners performed calibration exercises on subjects not associated with the study. Intra-rater correlations for CAL measurements ranged from 0.67 to 0.89 and inter-rater correlation was 0.70. For PD measurements, the examiners yielded intra-rater correlations between 0.68 and 0.88 and an inter-rater correlation of 0.72.

SHIP-START-2 and SHIP-TREND-0 were approved by the ethics committee of the University of Greifswald (SHIP-START-2 and SHIP-TREND-0: BB 39/08a issued on September 3rd, 2009). Written informed consent was obtained from all participants prior to their enrolment. The recommendations of the Strengthening the Reporting of Observational Studies in Epidemiology (STROBE) guidelines for observational studies were applied for reporting (von Elm et al., 2014).

Periodontal Examinations

Probing depth (PD), clinical attachment levels (CAL), and bleeding on probing (BOP) were assessed using a manual periodontal probe (SHIP-START-2: PCP 11; SHIP-TREND-0: PCPUNC 15; Hu-Friedy, Chicago, IL, USA). Measurements were performed at four sites per tooth (distobuccal, midbuccal, mesiobuccal, and midlingual/midpalatinal), excluding third molars. A half-mouth examination was conducted, with alternating quadrants in SHIP-START-2 and randomly selected quadrants in SHIP-TREND-0. PD was measured as the distance between the pocket base and the free gingival margin, while CAL was defined as the distance between the cemento-enamel junction (CEJ) and the pocket base. If the CEJ was not identifiable due to defects or restorations, CAL was not recorded. All measurements were rounded to the nearest whole millimeter. BOP was recorded immediately after probing at four sites (central incisor, canine, and first molar in each examined quadrant). If a reference tooth was missing, the next distal tooth was evaluated.

For sub-study I, the following periodontal parameters were used for the analysis: the percentage of sites with BOP (%BOP), the number of sites with PD ≥4 mm (PD≥4mm), mean PD, and the cumulative sum of all PD values (cumPD).

For sub-study II, the following periodontal parameters were derived for definition of periodontitis cases and periodontally healthy controls: the percentage of sites with BOP, the number of sites with PD ≥5 mm, the percentage of teeth with PD ≥4 mm.

Saliva sampling

Samples were taken between 8 a.m. and 3 p.m. prior to dental examinations. Stimulated saliva samples were collected using a Salivette (Sarstedt, Nürnberg, Germany). Briefly, participants were asked to chew a cotton roll for one minute, which was then inserted into the Salivette device and centrifuged (11,600 g, 20 min 4^o^C) which yielded clear saliva. To avoid protein degradation, protease inhibitor cocktail (v/v 1:5000, Sigma-Aldrich, St.Louis, MO, U.S.A.) was added (Jehmlich et al., 2013).

Due to the large number of samples, the preparations were divided into batches and processed according to the protocols described earlier(Murr et al., 2017). As described earlier (Jehmlich et al., 2013), a 500 µl aliquot per sample underwent precipitation with trichloroacetic acid (TCA) at a final concentration of 10% (v/v) in the presence of 0.12% (w/v) dithiothreitol. After incubation for 15 minutes, samples were centrifuged (16,200 g, 15 min, 4^o^C) and the protein pellets washed twice with 100% ice-cold acetone. The remaining protein pellet was reconstituted in 8M urea/ 2M thiourea Bradford assay (Bio-Rad, Hercules, CA) was used to determine protein concentrations.

A total amount of 3 µg protein per sample was reduced with 2.5 mM DTT (dithiothreitol) at 60°C for 60 minutes and alkylated with 10 mM iodoacetamide at 37°C for 30 minutes. Tryptic digestion was performed overnight at 37°C by adding trypsin to each sample at a protease to protein ratio of 1:25. Subsequently, digestion was stopped with acetic acid (final concentration 1% v/v) and peptides were purified on C18 material (μC-18 ZipTip) (Murr et al., 2017).

LC-MS/MS Analyses

Samples of **sub-study I** were measured in data-dependent mode on a nano-UPLC (nanoAcquity UPLC system, Waters, Milford, MA, U.S.A.) coupled to an LTQ-Orbitrap Velos mass spectrometer (Thermo Fisher Scientific, Bremen, Germany) as described earlier (Jehmlich et al., 2013). Protein identification and quantification were performed using the Genedata Expressionist Refiner MS software (Genedata AG, Basel, Switzerland) as described earlier (Murr et al., 2017). Briefly, detected ion clusters were searched against a human Uniprot database (v 12/2014) using the Mascot algorithm v2.4. Only proteins identified by at least two unique peptides and present in at least 40% of all samples were considered for further analysis. Protein intensities were median-median normalized and adjusted for batch and order of measurement in the linear regression model.

For **sub-study II,** LC-MS^E^-analysis was carried out on a nano UPLC (Acquity UPLC system, Waters) coupled to a Synapt G2-Si mass spectrometer (Waters). Peptide solution (≙ 400 ng) was loaded directly onto a nanoAcquity HSS T3 C18 analytical column and analysed in data-independent acquisition mode. Mass data was collected by alternating low and high energy modes of the collision cell. LC–UDMS^E^ data processing was performed in ProgenesisQI software (Nonlinear Dynamics, Durham, NC, USA) for automated peak picking and alignment. Data were searched against an Uniprot database (08/2013) limited to human entries, and only non-conflicted, proteospecific peptides were accepted for further analysis. Detailed LC-MS/MS and search parameters are summarized in Tables S5 and S6. Data are available via ProteomeXchange with identifier PXD006367 and PXD068002. Different false discovery rate (FDR) thresholds reflect platform-specific acquisition modes and established software-specific conventions rather than differences in analytical stringency.

Covariates

Trained and certified personnel conducted computer-assisted personal interviews to assess relevant covariates. The recorded variables included school education status (<10, 10, or >10 years), smoking status (never, former, or current smoker), tooth brushing frequency (<2 vs. ≥2 times daily), and utilization of dental services (at least one dental appointment in the past 12 months; yes/no). Additionally, standardized measurements of body height and weight were taken using calibrated scales, and the body mass index (BMI) was calculated (kg/m^2^). Blood samples were taken from the cubital vein of participants in the supine position. HbA1c was measured by high-performance liquid chromatography with spectrophotometric detection (Diamat Analyzer; Bio-Rad, Munich, Germany).

Statistical analyses

Continuous data are presented as means and standard deviations (SD); categorical data are presented as numbers (percentages).

**Sub-study I:** Associations between dental variables and protein levels of study participants (independent samples; independence of errors) were analysed using linear regression models. Continuous covariates such as age, BMI, and HbA1c, as well as categorical covariates such as sex, education level, smoking status, and tooth brushing frequency, were considered. We checked model assumptions graphically and using ANOVA models (approximate normal distribution of residuals; homoscedasticity; linearity of associations). Multicollinearity was checked via variance inflation factors and was found to be non-present. Beta coefficients and 95% confidence intervals (CI) were reported. To account for multiple testing (separately for each of the exposure variables), we adjusted the p-values from regression models by controlling the false discovery rate (FDR) at 10% using the Benjamini–Hochberg procedure (q value).

**Sub-study II:** Differences in abundance levels between periodontally healthy and diseased individuals were analysed separately for two age groups using two-sided t-tests. Fold change is reported.

Importantly, false-positive propagation was minimized by requiring independent replication across two sub-studies prior to downstream drug target screening.

Bioinformatics analysis

Bioinformatic analyses were performed to support biological interpretation rather than discovery. Functional enrichment analysis (p<0.05) was carried out employing g:Profiler, version *e113_eg59_p19_f6a03c19*, database updated on 23/05/2025 (Kolberg et al., 2023), specifically via the gprofiler 2 R package (Kolberg et al., 2020). To visualize molecule centered as well as pathway centered data, results for GO terms “biological process” and “molecular function” as well as the Reactome output are shown.

Targets and Drug banks

To identify pharmacologically actionable targets, we applied a drug repurposing strategy based on established pharmacological knowledge bases. Protein targets were queried across multiple curated platforms, including the Therapeutic Target Database (TTD), Open Targets (OT), and DrugBank (DB), which are commonly used resources in computational drug repurposing.

In addition to database-based screening, literature-based text mining was performed to capture reported drug–target associations beyond formal database annotations. For this purpose, Perplexity was used to systematically query the biomedical literature using disease- and target-specific prompts (e.g., “Which drugs could be used for anti-inflammatory diseases targeting protein XY?”). Retrieved information was manually reviewed to ensure biological relevance and contextual consistency.

Identified drug–target associations were subsequently filtered according to therapeutic area and biological plausibility. Only drugs targeting host-related pathways were considered, whereas compounds primarily affecting the microbiome were excluded. The combined results from curated databases and literature-based text mining were compiled to provide a comprehensive overview of proteins linked to approved drugs or clinically characterized compounds.

Protein selection and drug target screening

To be included in the drug target screenings, the proteins had to fulfil the following requirements: A) stringent analysis: i) **sub-study I**: proteins were required to be associated with at least two periodontal parameters (the percentage of sites with BOP (%BOP), the number of sites with probing depth ≥4 mm (PD≥4mm), mean PD, and the cumulative sum of all PD values) at a false discovery rate–adjusted q-value <0.05. ii) **sub-study II**: independent replication was defined by p-value <0.05 and an absolute fold change (FC) >1.5 between periodontally healthy controls and periodontitis cases within either the younger (35-44 years) or the older age group (55-64 years).  B) lenient analysis: i) sub-study I: proteins with q value <0.1 in the linear regression analysis for any of the four periodontal variables (meanPD, cumPD, PD≥4mm or %BOP), ii) sub-study II: proteins that differed significantly (p value <0.05, *abs*(FC) >1.3) between periodontally healthy controls and periodontitis cases within either the younger (35-44 years) or the older age group (55-64 years).

Results

Sub-study I - Baseline characteristics

SHIP-TREND-0 participants were on average 49.6 years old and 51.1% were male (Table 1). Thirty-seven percent had more than 10 years of education, 25% were current smokers. Their average BMI was 27.5 (SD 4.3); 46% and 24.1% of participants were overweight and obese, respectively. On average, %BOP was 21.8% (SD 23.5), mean PD was 2.45 mm (SD 0.52), and PD≥4mm was 4.3 (SD 5.8).

Sub-study I – Enrichment analysis

Functional analysis of these 67 proteins was carried using g:Profiler (Figure S1, Table S2). Regarding Gene Ontology:Biological Processes (GO:BP), proteins were categorized based on their role in the response to biotic stimulus, including antibacterial humoral response. Concerning Gene Ontology:Molecular Function (GO:MF), proteins were assigned to categories of endopeptidase activity. Functional pathway analysis using the Reactome database (REAC) revealed categories associated with innate immune system, with a particular emphasis on neutrophil-related pathways and platelet degranulation. These results directly reflect the central inflammatory cascade triggered by periodontitis.

Sub-study II - Baseline characteristics

In both age groups of sub-study II, periodontally healthy controls exhibited a lower risk profile than periodontitis cases (Table S3). Specifically, they had a higher level of education, were more often never-smokers, and had a lower average BMI. Distributional differences in periodontal variables (%BOP, maximum PD, number of sites with PD ≥5 mm, the percentage of teeth with PD≥4mm) were controlled by the study design.

Sub-study II – Enrichment analysis

In sub-study II, enrichment analyses of proteins differentially abundant between periodontitis cases and healthy controls identified functional patterns largely consistent with those observed in sub-study I (**Figure 4A, Figure S1, Table S2**), despite differences in study design and age stratification. Enriched **GO:BP** terms reflected sustained immune activation and stress responses, including pathways linked to neutrophil function, oxidative stress regulation, and cellular detoxification. **GO:MF** categories related to antioxidant activity and proteolysis further emphasized the involvement of redox balance and tissue remodeling. **REAC** pathway analysis confirmed enrichment of immune system–related pathways across both age groups, with minor variations in pathway prominence, supporting biological convergence between case–control status and continuous measures of periodontal inflammation.

All analyses presented in this supplement are exploratory and intended to support biological interpretation rather than causal inference.

References

Jehmlich, N., Dinh, K. H., Gesell-Salazar, M., Hammer, E., Steil, L., Dhople, V. M., Schurmann, C., Holtfreter, B., Kocher, T., & Volker, U. (2013). Quantitative analysis of the intra- and inter-subject variability of the whole salivary proteome. *J Periodontal Res*, *48*(3), 392-403. <https://doi.org/10.1111/jre.12025>

Keil, U., Stieber, J., Doring, A., Chambless, L., Hartel, U., Filipiak, B., Hense, H. W., Tietze, M., & Gostomzyk, J. G. (1988). The cardiovascular risk factor profile in the study area Augsburg. Results from the first MONICA survey 1984/85. *Acta Med Scand Suppl*, *728*, 119-128. <https://doi.org/10.1111/j.0954-6820.1988.tb05563.x>

Kolberg, L., Raudvere, U., Kuzmin, I., Adler, P., Vilo, J., & Peterson, H. (2023). g:Profiler-interoperable web service for functional enrichment analysis and gene identifier mapping (2023 update). *Nucleic Acids Res*, *51*(W1), W207-W212. <https://doi.org/10.1093/nar/gkad347>

Kolberg, L., Raudvere, U., Kuzmin, I., Vilo, J., & Peterson, H. (2020). gprofiler2 -- an R package for gene list functional enrichment analysis and namespace conversion toolset g:Profiler. *F1000Res*, *9*. <https://doi.org/10.12688/f1000research.24956.2>

Murr, A., Pink, C., Hammer, E., Michalik, S., Dhople, V. M., Holtfreter, B., Volker, U., Kocher, T., & Gesell Salazar, M. (2017). Cross-Sectional Association of Salivary Proteins with Age, Sex, Body Mass Index, Smoking, and Education. *J Proteome Res*, *16*(6), 2273-2281. <https://doi.org/10.1021/acs.jproteome.7b00133>

Volzke, H., Schossow, J., Schmidt, C. O., Jurgens, C., Richter, A., Werner, A., Werner, N., Radke, D., Teumer, A., Ittermann, T., Schauer, B., Henck, V., Friedrich, N., Hannemann, A., Winter, T., Nauck, M., Dorr, M., Bahls, M., Felix, S. B., . . . Kocher, T. (2022). Cohort Profile Update: The Study of Health in Pomerania (SHIP). *Int J Epidemiol*, *51*(6), e372-e383. <https://doi.org/10.1093/ije/dyac034>

von Elm, E., Altman, D. G., Egger, M., Pocock, S. J., Gotzsche, P. C., Vandenbroucke, J. P., & Initiative, S. (2014). The Strengthening the Reporting of Observational Studies in Epidemiology (STROBE) Statement: guidelines for reporting observational studies. *Int J Surg*, *12*(12), 1495-1499. <https://doi.org/10.1016/j.ijsu.2014.07.013>

Supplementary Figures

**
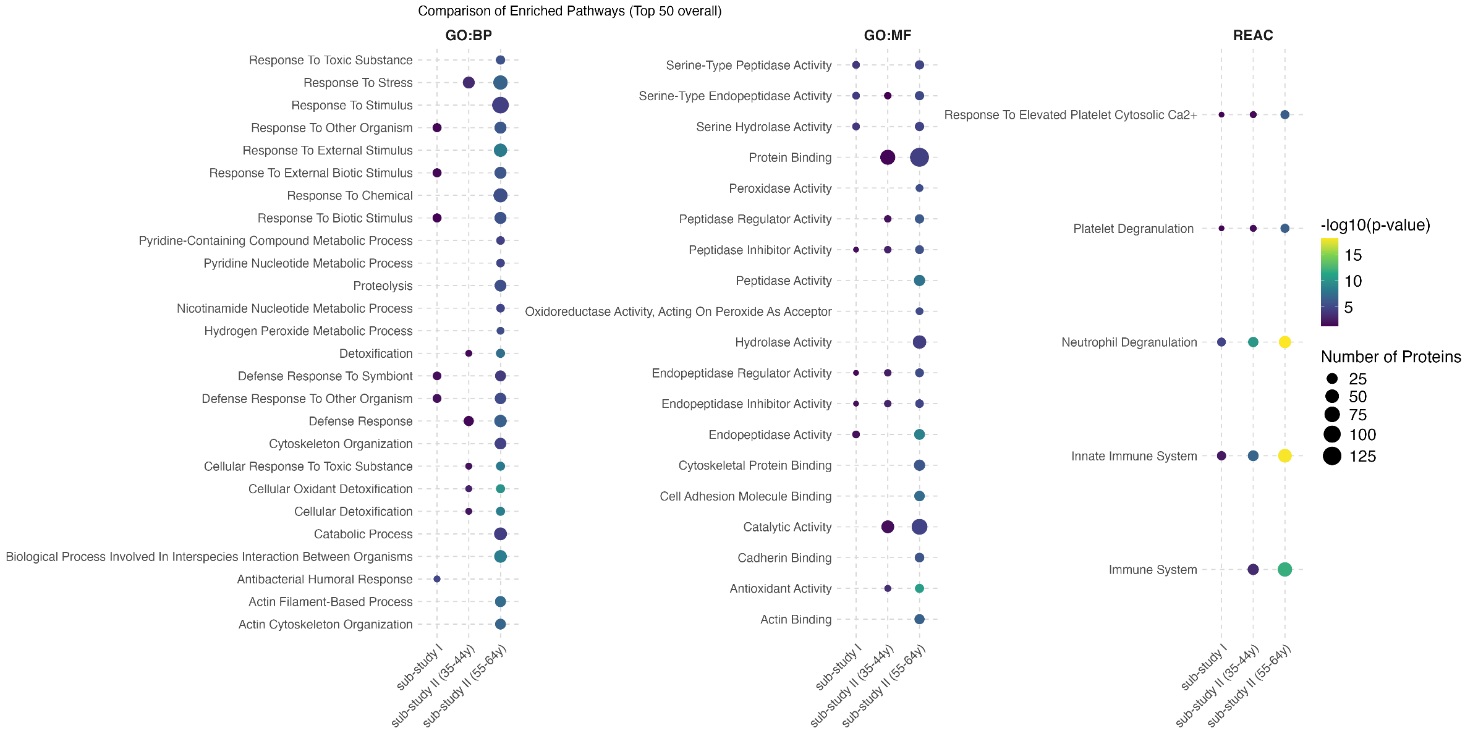
**

**Figure S1. Comparison of protein enrichment across different studies for biological processes (GO:BP), molecular functions (GO:MF), and Reactome (REAC) pathways.** The dot plot visualizes the Top50 significantly enriched terms for biological processes (GO:BP), molecular functions (GO:MF), and Reactome (REAC) pathways. The size of each dot is proportional to the number of proteins associated with a given term, while the dot colour represents the significance of the enrichment, shown as the negative log-transformed p-value.

Supplementary Tables

**Table S1:** Detailed results from linear regression analyses for sub-study I.

**Table S2:** Detailed Results of Protein Enrichment Analysis (GO and Reactome).

**Table S3:** Results from analysis of younger (33-45 years) and older (55-64 years) age groups in sub-study II.

**Table S4:** Functional annotation of the 26 target proteins identified across both sub-studies.

**Table S5**. Detailed LC-MS/MS and database search parameters for measurement and database search for sub-study I (Murr et al., 2017).

| **Reversed Phase Liquid Chromatography (RPLC)** |  |
| --- | --- |
| Nano-LC | nanoAcquity UPLC system, Waters |
| trap column | nanoAcquity symmetry C18 (2G-V/M, 2 cm, 180 µm ID, 5 µm C18) |
| analytical column | 10 cm nanoAcquity Peptide BEH C18 (100 µm ID, 1.7 µm C18) |
| buffer system | 0.1 % acetic acid, 2 % DMSO, 2 % ACN in water (buffer A) and  0.1 % acetic acid, 5 % DMSO in ACN (buffer B) |
| flow rate | 400 nL/min |
| gradient | 5 % to 25 % buffer B over a time of 63 min |
| column oven temperature | 40 °C |
| **Mass Spectrometry (MS)** |  |
| MS system | LTQ Velos Orbitrap, Thermo Electron |
| data acquisition mode | data-dependent |
| ion source | nanoelectrospray ion source (PicoTip Emitter, New Objective, Woburn, MA, USA) |
| **full MS** |  |
| resolution | 30,000 |
| scan range | 300 to 1,700 m/z |
| **ddMS2** |  |
| selection for MS/MS | Top20 |
| Selected charge states | 2 and 3 |
| MS/MS per feature (Repeat count) | 1 |
| fragmentation | collision-induced dissociation |
| collision energy | 35 % |
| dynamic exclusion | 60 s |
| min. MS/MS ion signal | 2,000 |
| **Database Search (Mascot)** |  |
| database | Uniprot/Swiss-Prot, only human entries (version 2014-12, 20,193 entries) |
| parent mass tolerance | 10 ppm |
| fragment ion mass tolerance | 0.6 Da |
| fixed modification | carbamidomethylation (cysteine) |
| variable modification | oxidation (methionine) |
| false positive rate | 1 % (target-decoy) |

**Table S6**. Parameters for measurement and database search for sub-study II.

| **Reversed Phase Liquid Chromatography (RPLC)** |  |
| --- | --- |
| Nano-LC | MDLC, Waters |
| trap column | NA |
| analytical column | nanoAcquity HSS T3 C18 analytical column (20 cm, 75 µm I.D., 1.7 µm particle size) |
| buffer system | 0.1 % acetic acid, 2 % ACN in water (buffer  A) and  0.1 % acetic acid in ACN (buffer B) |
| flow rate | 300 nL/min |
| gradient | 3 % to 35 % buffer B over a time of 120 min |
| column oven temperature | 45 °C |
| **Mass Spectrometry (MS)** |  |
| MS system | Synapt G2-Si (Q-TOF) |
| data acquisition mode | data-independent (MSe) |
| ion source | Nanno LockSpray source |
| Ion mobility separation | yes |
| T-wave velocity ramp | 800-500 m/s, 40 V |
| Lock spray | GluFib precursor 785.8426 m/z |
| **Full MS** |  |
| resolution | 28, 000 |
| scan range | 50 to 2000 m/z |
| Low energy scan | 4 eV |
| High energy scan | 25-55 eV |
| Cycle time | 1 sec |
| **Peak picking and alignment** |  |
| Software peak processing | ProgenesisQI v2.0 |
| Charge state | 2-6 |
| Noise estimation | default |
| Chromatographic peak width | not defined |
| Alignment | automatic alignment by retention time |
| Review alignment | Yes (>90%) |
| **Database Search/Quantification** |  |
| Software database search | MSE built in search engine of Progenesis QI v2.0 |
| Algorithm | Ion accounting search |
| database | Uniprot/Swiss-Prot, only human entries (version 2013-08, 20,235 entries) |
| fixed modification | carbamidomethylation (cysteine) |
| variable modification | oxidation (methionine) |
| Missed cleavages | 1 |
| fragment ions per peptide | 2 |
| fragment ions per protein | 5 |
| charge state | 2-6 |
| false positive rate (peptide ion level) | 4 % (target-decoy) |
| Peptide score threshold | 3 |
| Peptide filter | Non-conflicting peptides |
| Protein quantitation | Relative quantification using Hi-3 |
| Data transformation | Log10 transformation |
| Data normalization | Median normalization |
